# Facilitators and barriers to non-medical prescribing – A systematic review and thematic synthesis

**DOI:** 10.1371/journal.pone.0196471

**Published:** 2018-04-30

**Authors:** Emma Graham-Clarke, Alison Rushton, Timothy Noblet, John Marriott

**Affiliations:** 1 School of Pharmacy, Institute of Clinical Sciences, College of Medical and Dental Sciences, University of Birmingham, Birmingham, United Kingdom; 2 Centre of Precision Rehabilitation for Spinal Pain, School of Sport, Exercise and Rehabilitation Sciences, College of Life and Environmental Sciences, University of Birmingham, Birmingham, United Kingdom; Universidad del Desarrollo, CHILE

## Abstract

**Introduction:**

Non-medical prescribing has the potential to deliver innovative healthcare within limited finances. However, uptake has been slow, and a proportion of non-medical prescribers do not use the qualification. This systematic review aimed to describe the facilitators and barriers to non-medical prescribing in the United Kingdom.

**Methods:**

The systematic review and thematic analysis included qualitative and mixed methods papers reporting facilitators and barriers to independent non-medical prescribing in the United Kingdom. The following databases were searched to identify relevant papers: AMED, ASSIA, BNI, CINAHL, EMBASE, ERIC, MEDLINE, Open Grey, Open access theses and dissertations, and Web of Science. Papers published between 2006 and March 2017 were included. Studies were quality assessed using a validated tool (QATSDD), then underwent thematic analysis. The protocol was registered with PROSPERO (CRD42015019786).

**Results:**

Of 3991 potentially relevant identified studies, 42 were eligible for inclusion. The studies were generally of moderate quality (83%), and most (71%) were published 2007–2012. The nursing profession dominated the studies (30/42). Thematic analysis identified three overarching themes: non-medical prescriber, human factors, and organisational aspects. Each theme consisted of several sub-themes; the four most highly mentioned were ‘medical professionals’, ‘area of competence’, ‘impact on time’ and ‘service’. Sub-themes were frequently interdependent on each other, having the potential to act as a barrier or facilitator depending on circumstances.

**Discussion:**

Addressing the identified themes and subthemes enables strategies to be developed to support and optimise non-medical prescribing. Further research is required to identify if similar themes are encountered by other non-medical prescribing groups than nurses and pharmacists.

## Introduction

The drive behind non-medical prescribing in the United Kingdom (UK) is the need to deliver high-quality healthcare to patients where and when they require it, within a limited financial resource [1–3]. Innovative patient centred care pathways are being developed, using the most appropriate healthcare professionals, such as clinical pharmacists in general practice [4], or prescribing physiotherapists streamlining musculoskeletal pathways [5]. The extension of non-medical prescribing to other professional groups continues; with pressure for physician associates to become prescribers [6] and paramedics; who were unsuccessful at the last consultation [7].

Non-medical prescribing evolved from limited list prescribing for a few nurses in the early 1990s to the current range of eligible healthcare professionals (Table 1). Each healthcare professional must successfully complete an appropriate and approved prescribing course, and be registered as a prescriber with their relevant regulatory body. Professionally, they are expected to prescribe within their competency area [8, 9].

**Table 1:** Evolution of non-medical prescribing in the UK.

2002	Extended formulary prescribing for nurses
2003	Supplementary prescribing for nurses and pharmacists
2005	Independent prescribing for nurses and pharmacists Supplementary prescribing for physiotherapists, podiatrists, and therapeutic and diagnostic radiographers
2008	Independent prescribing for optometrists
2012	Independent prescribing for physiotherapists and podiatrists
2016	Independent prescribing for therapeutic radiographers Supplementary prescribing for dieticians

An independent prescriber is responsible for the care of the patient, including prescribing.

A supplementary prescriber works in collaboration with an independent prescriber and the patient to prescribe according to a pre-determined treatment scheme.

The initial uptake of non-medical prescribing was slow, with approximately 240 pharmacists and 4000 nurses having qualified by 2005 [10], the later contrasting with the government’s anticipated 10000 nurses [11]. A recent report identified that approximately 53000 nurses and over 3800 pharmacists were registered as prescribers in 2015 [12], but was unable to identify how many were active. Previous survey evidence indicated 14% of nurse independent prescribers and 29% of pharmacist independent prescribers were not using their prescribing qualification [10], and other estimates [13] indicate under 10% of nurse independent prescribers and nearly 40% of pharmacist and allied health professional prescribers are not using their prescribing qualification. Similarly, surveys conducted by the General Pharmaceutical Council indicate varying uptake of prescribing activity. In a 2016 survey of prescribing pharmacists nearly 90% of pharmacist prescribers were reported as active [14], whereas the previous 2014 report had found that only 61% had prescribed in the previous year [15]. The 2016 survey had a poor response rate (<18%) possibly overestimating activity through responder bias.

The full cost of training a non-medical prescriber (NMP) has been calculated as approximately £10000 [10] and, with increasing demand on the NHS and limited funding, there is a need to realise the full benefit of training investment. Previous studies have identified reasons for not prescribing including lack of support from colleagues or within their work environment [13, 14], or a role change [10]; but did not explore these issues in depth. A previous thematic literature review of supplementary prescribing did not address the issue of barriers and facilitators specifically, but identified a limited number including: medical practitioner support, communication, resource limitations and specific supplementary prescribing aspects [16]. It also did not address independent prescribing. There has been no robust review of the qualitative literature relating to barriers or facilitators of independent non-medical prescribing. Identifying facilitators and barriers to independent non-medical prescribing has the potential for strategy development to optimise its implementation.

The aim of this review was to evaluate the use, facilitators, and barriers of independent non-medical prescribing in primary and secondary care in the UK.

## Methods

### Search strategy and selection criteria

A systematic review and thematic synthesis was conducted to explore the barriers and facilitators to non-medical independent prescribing in the UK. A protocol for the review was developed in advance, following the PRISMA-P statement [17], and registered with PROSPERO (CRD42015019786). The results are reported in accordance with the PRISMA and ENTREQ statements (S1 and S2 Appendices) [18, 19].

Qualitative and mixed methods research studies investigating independent non-medical prescribing in the UK were included. Narrative reports describing a service, opinion papers and abstracts were excluded [20]. The legislation permitting independent prescribing by nurses and pharmacists was enacted in 2006 and therefore only studies published since 2006 were included [21]. There was no language restriction.

Specific search strategies were developed with expert librarian support, for each electronic database, and included broad and narrow, free text, and thesaurus based terms [22]. Boolean operators and truncation were used. The selected keywords were: nurse, pharmacist, physiotherapist, podiatrist, non-medical, therapist, allied health professional, chiropodist, independent prescribing, utilisation, barriers, facilitators, role, education, support, guidelines, policy, procedures, attitudes and clinic. The following databases were searched: AMED, ASSIA, BNI, CINAHL, EMBASE, ERIC, MEDLINE, Open Grey, Open access theses and dissertations, and Web of science. Papers that cite, or were cited by, the included papers were screened to identify any further relevant papers. Searches were completed to 26 March 2017 (S3 Appendix. Medline (Ovid) search strategy).

Titles/abstracts obtained from all searches were screened to remove duplicates and papers that did not meet the eligibility criteria. Full text copies of the papers remaining were obtained and reviewed. Two independent reviewers (EGC and TN) conducted each stage and resolved differences by discussion, with a third reviewer (AR) available for mediation if required [23]. Numbers excluded at each stage were recorded [18, 23].

### Quality assessment

A validated quality assessment tool, (Quality Assessment Tool for Studies of Diverse Designs, QATSDD), was used to assess the studies [24]. The tool was developed to support quality analysis where studies use different designs, including qualitative, quantitative, and mixed methods. The tool comprises 16 elements (listed in S1 Table. QATSDD scores for each paper) covering aspects such as theoretical approach, research setting, data collection, and method of analysis. Each element is rated on a scale of 0 –no evidence, to 3 –full details, with clear reasons defined for each score. Twelve elements are common to all studies, with two specific elements each for qualitative and quantitative studies. The studies included in this review used a variety of research methods, primarily interviews, questionnaires and focus groups, making this tool suitable. Two reviewers (EGC and TN) independently assessed the studies using the tool; resolving any disagreement in the scores through discussion. Including low quality studies in a qualitative systematic review is debated, with some researchers arguing for their inclusion as they may provide valuable insights, whereas others argue they should be excluded [20, 25, 26]. The decision was taken to include all studies to inform synthesis and conclusions regardless of quality assessment, but to report on the quality assessment results (see Table 2), particularly as from an initial scoping search, limited studies were identified.

**Table 2:** Characteristics and details of selected papers.

Author	Population	Setting and/or speciality	Study type	Participant numbers	Results/Findings	QATSDD
Adigwe (2012) [27]	NMPs Patients	Primary & secondary care	1) SSI-F2F 2) Online survey 3) SSI-F2F	1) NP (n = 9) PP (n = 13) 2) NP (n = 141) PP (n = 27) 3) Other NMP (n = 11) Patients (n = 12)	Supportive mechanisms & safe prescribing environment required to support prescribers	90%
Armstrong (2015) [28]	Senior nurse Medical consultant NP Nurse Pharmacist Patients	Urgent care setting—one hospital	1) SSI 2) Questionnaire	1) Senior nurse (n = 1) Doctor (n = 1) NP (n = 2) Nurse (n = 1) Pharmacist (n = 1) 2) Patients (n = 20)	Benefits of autonomous working identified by staff & patients.	45%
Bennett et al (2008) [29]	Practising NP	HIV clinics—community & secondary care	1) postal questionnaire 2) Focus group	1) NP (n = 8) 2) NP (n = 7)	Impact of prescribing on NP/doctor and patient relationships discussed. Overall perceived to be beneficial.	45%
Bewley (2007) [30]	Recently qualified nurses Senior paediatric nurses NP HEI	Paediatrics	1) Facilitated workshop 2) Facilitated workshop 3) Narrative 4) Semi-structured questionnaire 5) Scoping exercise	1) Recently qualified nurses (n = 35) 2) Senior paediatric nurses (n = ?) 3) NP (n = 1) 5) NP (n = 19) 5) HEI (n = 4)	Pharmacology knowledge poor during nurse training. Identified as challenging in NMP course.	14%
Bowskill (2009) [31]*	NP	Primary & secondary care	SSI	NP (n = 26)	Trust between nurse and doctor identified as necessary for a successful prescribing partnership.	90%
Bowskill et al (2013) [32]*	NP	Primary & secondary care	SSI	NP (n = 26)	Trust between nurse and doctor identified as necessary for a successful prescribing partnership. Secondary care practitioners had more restrictions.	60%
Brodie et al (2014) [33]	PP NP	Primary care	SSI-F2F	PP (n = 4) NP (n = 4)	PP/NP have holistic approach to treatment. Concerns they were underutilised.	38%
Carey et al (2009) [34]†	NP	Specialist children’s hospital—Intrinsic case study	Interviews	NP (n = 7 participants, 18 interviews)	NMP believed to improve care provided to patients.	55%
Carey et al (2009) [35]†	NP Doctors DMPs Clinical Leads	Specialist children’s hospital—Intrinsic case study	SSI-F2F	NP (n = 7 participants, 18 interviews) Doctors (n = 4) DMPs (n = 7) Clinical Leads (n = 3)	Successful NMP implementation but variations in approach and expectations.	48%
Carey et al (2010) [36]‡	NP Doctors Administration staff Non-nurse prescribers	Dermatology services—primary & secondary care– 10 site collective case study	SSI-F2F	NP(n = 11) Doctors (n = 12) Administration staff (n = 11) Non-nurse prescribers (n = 6)	NMP improved access to treatment, with ability for service reconfiguration. Inconsistent support post-training.	45%
Carey et al (2014) [37]	NP	Respiratory conditions - Primary & secondary care, East of England SHA	SSI—telephone	NP (n = 39 Non-prescribing NP (n = 1)	Wide variations in practice, but overall improved service to patients. Several challenges to NMP identified.	62%
Courtenay et al (2008) [38]	NP	Primary & secondary care	Questionnaire	NP (n = 1377)	Nearly 70% of NP reported problems with implementing NMP.	56%
Courtenay et al (2009) [39]†	Doctors DMPs Clinical leads	Specialist children’s hospital—Intrinsic case study	F2F interviews	Doctors (n = 7) DMPs (n = 4) Clinical leads (n = 3)	Benefits in improving services to patients identified, but concerns raised regarding roles and NMP selection.	71%
Courtenay et al (2009) [40]‡	NP Doctors Administration staff Non-nurse prescribers Patients	Dermatology services—primary & secondary care– 10 site collective case study	1) SSI-F2F 2) Videotaped observations 3) Questionnaire	1) NP (n = 10) Doctors (n = 12) Administration staff (n = 11) Non-nurse prescribers (n = 6) 2) NP (n = 37) 3) Patients (n = 165)	Benefit to care reported by patients.	56%
Courtenay et al (2011) [41]	NMP leads, of whom half had a prescribing qualification	Primary & secondary care—one SHA	SSI	NMP leads (n = 28)	Four key aspects of role identified: information, promotion, clinical governance, and training	52%
Cousins et al (2012) [42]	NP	General practice	SSI-F2F	NP (n = 6)	NMP enhanced job satisfaction, but increased work-related stress.	57%
Dapar (2012) [43]	PP	Community, primary & secondary care	1) Questionnaire 2) Telephone interview	1) PP (n = 695/1643) 2) PP (n = 34)	Implementation of NMP requires support, and ability to overcome challenges. NMP role clarification required.	98%
Daughtry et al (2010) [44]	NP	One PCT, north England	SSI	NP (n = 8)	NMP expands role, but misunderstandings exist with other work colleagues.	38%
Dobel-Ober et al (2010) [45]	Nursing directors	Mental health trusts—England	Postal questionnaire	Directors of nursing (n = 39/66)	Majority of trusts had policies and strategies supporting NMP. Only 1 Trust had no NMPs.	46%
Downer et al (2010) [46]	NP	Community—two health boards, Scotland	Conversational F2F interviews	NP (n = 8)	Benefits to self and patients identified, but also challenges, including lack of support.	48%
Green et al (2008) [47]	NP (n = 12) PP (n = 1)	Mental health trust—Humber	Email qualitative survey	NMP (n = 10) (profession not indicated)	50% prescribing, others providing advice. NMP qualification of positive benefit.	48%
Herklots et al (2015) [48]	NP	Community—two PCTs	SSI	NP (n = 7)	NMP enhanced role, and knowledge from course beneficial to wider practice. Support, inc. CPD, variable.	50%
Hill et al (2014) [49]	Patients PP GPwSI	Addiction services—Lanarkshire	1) SSI based on questionnaire 2) Questionnaire alone	1) Patients (n = 86) PP (n = 5) 2) GPwSI (n = 6)	Overall satisfaction with PP led clinic, with enhanced job satisfaction.	33%
Kelly et al (2010) [50]	Practice nurses, +/- prescribing qualification	Primary care—one southern English county	Postal questionnaire	No prescribing qualification (n = 120) NP (n = 31)	46% respondents not intending to train as NMP, citing various issues relating to the course and age as reasons	35%
Maclure et al (2013) [51]	General public	Scotland	Postal questionnaire	General public (n = 1855/5000)	General support for NMP, but several concerns raised.	43%
Maddox (2011) [52]§	PP NP	Primary & community—predominantly NW England	1) Unstructured interviews 2) Focus group x 3 3) SSI (F2F or telephone) 4) Q-method	1) PP (n = 4) NP (n = 14) 2) NP (n = 10) 3) PP (n = 5) NP (n = 15) 4) PP (n = 22) NP (n = 34)	NMPs most confident when prescribing within guidelines. ‘Time burden’ for DMPs acknowledged as significant.	95%
Maddox et al (2016) [53]§	PP NP	Primary & community—predominantly NW England	1) SSI (F2F or telephone) 2) Focus group x 3	1) PP (n = 5) NP (n = 15) 2) NP (n = 10)	NMPs cautious when prescribing, confidence improved with good support.	69%
McCann et al (2011) [54]¶	PP	Primary & secondary care—Northern Ireland	Postal structured self-administered questionnaire	PP (n = 76/100)	Over 50% had or were not prescribing. Issues included lack of funding and lack of GP awareness.	42%
McCann et al (2012) [55]¶	PP DMP Key stakeholders	Primary & secondary care—Northern Ireland	SSI-F2F	PP (n = 11) DMP (n = 8) Stakeholders (n = 13)	Benefits of holistic care for patient and team working identified, together with several challenges.	60%
McCann et al (2015) [56]¶	PP Patients	3 case studies, primary & secondary care—Northern Ireland	Focus Groups x 7	Patients (n = 34)	Lack of prior awareness of PP. Patients identified benefits of team approach, but expressed some reservations.	62%
Mulholland (2014) [57]	PP Non-prescribing pharmacists	Neonatal units, United Kingdom	Electronic survey	PP (n = 22) Non-prescribing pharmacists (n = 23)	NMP identified as a team benefit, with utilisation of pharmacist knowledge.	23%
Mundt-Leach (2012) [58]	NP	NHS addiction services	Telephone survey	NP (n = 20)	Benefits of NMP for patients felt to outweigh challenges.	21%
Oldknow et al (2010) [59]	NP Consultant psychiatrists Patients	Older peoples’ mental health services—one mental health trust	1) F2F interviews 2) Postal survey 3) Document review	1) Participants unknown (n = ?) 2) Patients (n = 16/58) 3) Unknown	Report of a pilot implementation of NMP, which indicated service benefits.	35%
Oldknow et al (2013) [60]	Non-prescribing NP	One mental health trust	Interviews	Non-prescribing NP (n = 6)	Several barriers identified, including lack of remuneration.	71%
Ross (2015) [61]	NP PP Nurse manager Consultant psychiatrists GP Patients	Mental health—Tees, Esk & Wear Valleys NHSFT	1) Focus groups x 9 2) Interviews—F2F & telephone (n = 13)	1) & 2) Distribution unknown. NP (n = 35) PP (n = 3) Nurse manager (n = 2) Consultant psychiatrists (n = 7) GP (n = 1) Patients (n = 9)	Patient/NP relationship positive with benefit seen by all participants. De-prescribing highlighted as an important role.	60%
Ross et al (2012) [62]	NP	Mental health—Scotland	3) Email/postal Questionnaire 4) Focus group	1) NP (n = 33/60) 2) NP (n = 12)	Majority of NMPs yet to prescribe. Numerous barriers identified including lack of support from employer and lack of adequate remuneration.	71%
Shannon et al (2011) [63]	GP Cardiac physician	Heart Failure—one primary care centre & one hospital, West Scotland	1) Focus groups x 4 2) 1-2-1 interviews	1) GP (n = 9) Cardiac physician (n = 8) 2) GP (n = 1) Cardiac physician (n = 3)	Participants generally supportive of NMP, but identified communication as a key challenge.	57%
Stenner et al (2007) [64]‖	NP	Acute, chronic & palliative pain—community, primary & secondary care	SSI-F2F	NP (n = 26)	NMPs more likely to provide advice on treating chronic pain patients than prescribe. Reasons for this include budgetary restrictions.	57%
Stenner et al (2008) [65]‖	NP	Acute, chronic & palliative pain—community, primary & secondary care	SSI-F2F	NP (n = 26)	Many benefits to NMP identified, resulting from autonomous practice.	52%
Stenner et al (2008) [66]‖	NP	Acute, chronic & palliative pain—community, primary & secondary care	SSI-F2F	NP (n = 26)	Multi-disciplinary team working benefits both NMPs and other team members. Support from policies and CPD identified as important.	67%
Stenner et al (2010) [67]	NP Doctors Administration staff Non-prescribing nurse	Diabetes—community, primary & secondary care—9 site collective case study	SSI	NP (n = 10) Doctors (n = 9) Administration staff (n = 9) Non-prescribing nurse (n = 3)	Prescribing incorporated into existing role, with support from other staffs. Some issues initially, but now mainly resolved.	50%
Stenner et al (2011)[68]	Patients	Diabetes—6 sites, Primary care	SSI	Patients (n = 41)	Patients identified a range of benefits from NMP, including improved disease management.	57%

* paper derived from linked theses.

§ paper derived from linked theses.

† linked reports of data from one study.

‡ linked reports of data from one study.

¶ linked reports of data from one study.

‖ linked reports of data from one study.

DMP, designated medical practitioner; F2F, Face-to-Face; GP, general practitioner; GPwSI, GP with a special interest; HEI, Higher education institute; NHSFT, National Health Service Foundation Trust; NP, nurse prescriber; NMP, non-medical prescriber; PP, pharmacist prescriber; PCT, primary care trust; QATSDD, Quality Assessment of Studies of Diverse Designs; SHA, strategic health authority; SSI, Semi-Structured interviews

### Analysis

Thematic analysis, to identify recurrent barriers and facilitators to non-medical prescribing and themes relating to use, was conducted on text from the results and findings sections of the papers together with any included participant quotations [69, 70]. The studies were read to identify initial emerging themes, and then underwent line by line thematic coding utilising NVivo®11 (QSR International). As further themes emerged, new codes were created. All codes and themes were reviewed iteratively for consistency and appropriateness and amended if necessary. The findings were summarised under descriptive theme headings, permitting development of a hierarchy. The analysis was conducted by one researcher (EGC) and the initial themes and coding discussed and critically debated by all authors. The final version was agreed by all authors following further refinement of the theme headings and hierarchy. At the end of data analysis no further themes were identified, indicating that data saturation had been reached [70]. EGC is a practising NMP, and an NMP lead with a role in supporting other NMPs. This researcher standpoint was balanced by the other three authors, none of whom are prescribers.

## Results

**Fig 1:**
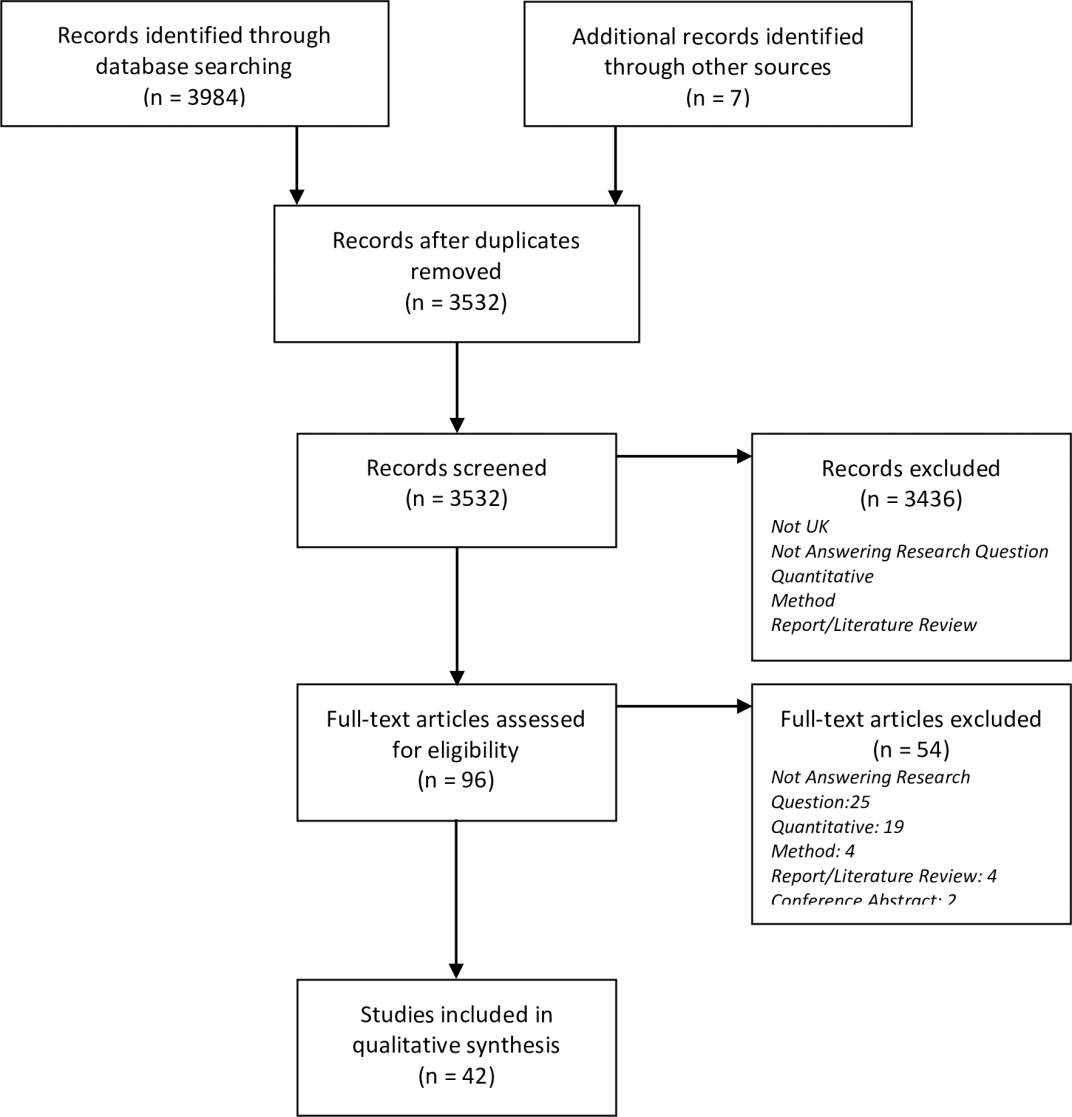
PRISMA flow diagram.

The search strategy identified 3991 potentially relevant studies. Following exclusion of 459 duplicates and 3436 from title and abstract review, 96 studies were reviewed at the full text stage. Following exclusions, 42 papers were included (Fig 1. PRISMA flow diagram).

Overall, the studies were assessed as moderate quality. There were three low scoring papers [30, 57, 58] (score <25%), and four high scoring papers [27, 31, 43, 52] (score >75%); the latter being doctoral theses (S1 Table. QATSDD scores for each paper). Key issues highlighted by the scores were poor reporting of theoretical framework, data collection tool choice, analytical method justification, research question and analytical method fit, and user involvement.

Of the 42 papers, 30 (71%) were published between 2007 and 2012, with the remainder published subsequently. Nurse independent prescribers were studied in 24 papers [28–32, 34–38, 40, 42, 44, 46, 48, 50, 58–60, 62, 64–67], pharmacist prescribers in five papers [43, 49, 54, 55, 57], and a mixture of nurse and pharmacist prescribers in a further six papers [27, 33, 47, 52, 53, 61]. The remaining papers investigated the views of patients and staff associated with NMPs [39, 41, 45, 51, 56, 63, 68].

Thematic analysis identified 17 subthemes of which 15 described the factors that may impact on NMPs and two described the range of activity. These were grouped into three overarching themes, which were 1) factors relating to the NMP themselves, 2) human factors and 3) organisational aspects. The themes and subthemes are presented in Table 3, together with example factors, and S2 Table lists the papers that the themes were identified in. The 15 subthemes impacting on non-medical prescribing contained factors which could be barriers or facilitators; in many instances, this was dictated by circumstances.

**Table 3:** The themes and subthemes that influence non-medical prescribing.

Theme	Sub, and subsub, themes	Quotations	Interpretation/example factors
1. Non-medical prescriber	1.1. Attitude [27–29, 31–35, 37, 40, 42, 44, 46–50, 52, 53, 61, 65–67]	“I think it’s been a marvelous (sic) thing really and it’s been good, it’s good for my confidence, it’s given me a lot to think about. It’s given me a new string to a bow, it, keeps me interested.” [37] “it scares the hell out of me even though I am autonomous in my clinics. I still after doing a prescription have to get a GP to sign to check” [50]	Job satisfaction and confidence of the practitioners enhanced by non-medical prescribing. Lack of confidence and anxiety can prevent practitioner from using prescribing skills. Attitude towards NMP and role can be affected by views of others.
	1.2. Practice		
	1.2.1. Area of competence [27, 31–35, 37, 38, 40, 42– 44, 48–53, 55–58, 65–68]	“… with contraception I thought before I start initiating new pills I really want to do an update and I was encouraged to do that quickly. It has given me a lot more confidence to prescribe in that area” [27] “Some of our patients now would be more difficult to manage you know patients with other conditions like some of our anaemia patients as well as being renal are also oncology patients and that makes them a bit more awkward and those patients I would definitely refer before upping or decreasing a dose” [55]	Confidence gained by defined area of competence. Constraints of co-morbidity acknowledged, need to refer when outside, or perceived to be outside, competence area.
	1.2.2. Role [27, 28, 31–37, 42–44, 46, 47, 50, 52, 53, 55, 56, 58, 62, 63, 65– 67]	“Hospital trust G and primary care trust A agreed for the nurse specialist to run nurse led clinics in primary care settings. Her prescribing qualification has enabled the successful development of this new service for patients. Without a nurse prescriber in these posts a doctor is required to be present in the community to prescribe for patients accessing healthcare at this point. “I couldn’t do my role without nurse prescribing”“ [31] “I have to develop my own role; fighting to find a place in between GPs and prescribing nurses” [43]	Found to enhance existing roles. Success more likely where practitioner’s role well-defined or when role specifically designed to include prescribing. Success less likely when lack of role clarity, where role wasn’t valued or where organisational issues constrained role development.
2. Human factors	2.1. Patients [27–29, 43, 47, 49, 51, 56, 59, 61, 63, 67, 68]	“I think they (nurse prescribers) look at all the care. They will check that the drugs they have prescribed don’t clash with other things. They are interested in my home life. They sit down and take an interest so you don’t relapse.” [61] “My one (disadvantage) would be crossing the specialisms–crossing the illnesses. My experience here is in relation to diabetic management, but I would also like one that is appreciative of my overall (health)” [56]	Patients appreciate receiving holistic care and understandable information from NMPs. Concerns about communication with GP, and that NMP may have limited knowledge/ability to deal with complex issues.
	2.2. Staff		
	2.2.1. Managers [27, 29, 31–33, 35–37, 41, 43, 45, 46, 50, 52, 54, 60, 62, 66, 67]	“…I think the non-medical prescribing lead did a good job in setting it up initially …we are lucky in our Trust because the non-medical prescribing lead has driven it from the onset, he was one of the first supplementary prescribers and he has driven its right from the word go really and he has fought long and hard to get it recognized and that's why we are in the position that we are in now.” [27] “Management appeared threatened, hostile and jealous of my prescribing authority and it is extremely annoying that major decisions regarding nurse prescribing are made by people in management who know nothing about it” [62]	Development and implementation of NMP, enabled by managerial support, including strong strategic vision. Implementation of NMP hindered through lack of understanding or organisational unpreparedness by managers.
	2.2.2. Medical professionals [27–29, 31–39, 43, 44, 46–50, 52–55, 57, 59–63, 65–67]	“Team working gives you much more information about the patient, and it gives you much more support if you need it; and I have a good working relationship with the GPs … I have referrals from the practice nurse; I have referrals from the doctor …So I think the close working relationship in the team is the best part”[43] “Again my anxiety is largely for the nurses involved; it doesn’t seem at the moment clear, exactly what their responsibility is and if there is a mess up, who carries the can. I am not clear if a nurse prescriber prescribes something at a dreadfully wrong dose and somebody is harmed as a result, who carries the can. Is that my MDU subscription or is it a separate thing? I think those areas are something that to me are not entirely clear.”[39]	Doctors understanding and appreciating benefits of NMP role, including seeking advice. Lack of clarity over role boundaries and concern over loss of control.
	2.2.3. Peers [27, 28, 31, 32, 34–36, 38, 41, 43, 44, 46–49, 52, 53, 55, 57, 58, 62, 65–67]	“Long term trusting relationship of mutual respect between medical, nursing and other health care professionals and myself” [43] “I think as soon as they realize you can prescribe they expect you to be able to do exactly what doctors can do. They don’t understand your limitations and you can only work within the scope of your knowledge, and they expect you to sign repeat prescriptions, and send everybody through to you. So it can be quite difficult at times explaining to them.” [44]	Peer/NMP relationship providing mutual support and improving team working. Lack of understanding of NMP and/or antagonism hindering NMP.
3. Organisational aspects	3.1. Administration		
	3.1.1. Formulary [27, 29, 31–33, 36, 37, 43, 47, 48, 51, 52, 62, 64, 66]	“You do take each patient on their own merit but within that framework and if there wasn’t that framework I think I might be floundering a bit more” [31] “The clinic is actually limiting the range of non-HIV medications that I can prescribe, even if many of these agents prove very useful in treatment support aims.” [29]	Personal formulary used to define area of competence, and supported by national guidelines. Formulary restrictions derived from organisational policy or cost pressures.
	3.1.2. Policy [27, 28, 31, 36, 43, 47, 62, 66]	“I guess the only thing that I would change is by having standards across the country, I think each Trust is allowed to adopt non-medical prescribing within their own guidelines and within their remit and I think it's been good in some areas but it has hindered non-medical prescribing in some others and it has not allowed them to develop their practice, as they would do.” [27] “My Trust has no guidelines and there is no guidance. I don’t know anyone in our area who is prescribing” [62]	Clear policy supporting NMP, and acting as safeguard. Lack of, or restrictive, policy hindering NMP development and implementation.
	3.1.3. Remuneration [27, 37, 43, 46, 50, 54, 60, 62]	“…you know, at the end of the day, I am doing it not for the money and not for the banding, it is for my practice and having a qualification that allows me to develop my practice but also to manage my career plan for the future, if you like …” [27] “I think that if there was a clear reward in taking up the nurse prescribing mantel, you know, I would be prescribing now” [60]	Prescribing qualification for role extension or career progression, not for financial reward. Lack of financial reward seen as disincentive to training and unappreciative of role.
	3.2. Development		
	3.2.1. Post course support [27, 28, 33, 35–37, 39, 41, 43, 46–48, 50–53, 55, 57, 62, 63, 65–67]	“I support them to ensure that they have access to further training, development and [continuous professional development]” [28] “Ongoing support has gone very hit and miss. In the first year there were a few evening sessions on general stuff, not specific to dermatology. Now with all the reorganisation it has completely hit the bin and you don’t get any CPD from the employer.” [36]	Post training support necessary for continued development of skills and confidence. Enabled by provision of training courses, and managerial support. Time and funding provision limiting access to courses. Peer and professional support absent.
	3.2.2. Training [27, 28, 30, 33, 35, 39, 41, 43, 45–48, 50, 52, 53, 56, 57, 62, 63, 67]	“All candidates have been required to […] have some clear objectives around the need and use of the skills and ability to prescribe.” [45] “Nurses that have done course say [very] intense and difficult. I have two children and am single parent–so limited commitment to study” [50]	Prior to course, need for NMP should be identified, and appropriate candidates selected. Role of clinical mentor crucial for successful completion. Time and course commitments off-putting or leading to challenges in completing course.
	3.3. Service delivery		
	3.3.1. Impact on time [27–29, 33–37, 39, 40, 43, 44, 46–49, 51, 52, 55–59, 61, 63, 65, 67, 68]	“I think it’s because of timing issues, you know, because normally if it’s someone who has rung in the morning, then they won’t get a GP visit till the afternoon, and if they’re last on the list, by then they’re so far down the line they’re in hospital. So timing issues are very important in managing a deteriorating patient … you get it on board quicker; I mean, it’s a 12-hour difference sometimes.” [48] “Oh, it has changed dramatically. Workload had trebled. We see most of the minor ailments. We have taken a lot more on—the more knowledge you get the higher the workload. We do all medication reviews and all hypertension reviews” [44]	Patients able to receive timelier and streamlined care with NMP. Ability to prescribe saves time for NMP, doctor, and patient. Workload pressure increasing because of prescribing.
	3.3.2. Infrastructure [27, 31–34, 36–38, 43, 46–49, 51, 52, 54, 57, 62–64, 67]	“What we get on the referral is what we know. I think we’ve had three more practices now go on to the same system we’re on and the GPs are finally coming round to understanding that sharing their notes is a benefit to all of us. So it is improving. I’ve now got two [GP practices] on my caseload where I can see their notes as well.” [37] ‘‘I feel that pharmacy independent prescribing can only take place in a primary care setting, within GP practices. This is because we have no access to patient history and notes otherwise. This makes prescribing from elsewhere more difficult and possibly less effective” [54]	Prescribing supported by good access to patient records, particularly electronic systems. Limited or no access to patient records (including electronic) preventing prescribing and impeding good communication.
	3.3.3. Service [27–29, 33–37, 39–44, 47–49, 52, 53, 55–59, 61, 63, 65, 68]	“I can do their prescription there and then, whereas sometimes they’d have to come back for it. For the younger people, who have taken time off work, they don’t want to come back again, and sometimes they get angry or frustrated if it puts them out, so yes, it’s much, much better for them that it’s done there and then.” [37] “At the moment we only have one [nurse prescriber] so it makes it impossible if X is off sick for another nurse to do her clinic without a lot of stress for the other person. And also time consuming for the patients because that nurse might have all the knowledge and skills but they will have to get the doctor to come in because they have not done the prescribing course.” [36]	Service to patient improved and streamlined, with improved patient satisfaction and efficiency. Services dependent on NMPs, with issues arising when NMPs are unavailable.
	3.3.4. Use in practice		
	3.3.4.1. Patients [29, 31–37, 39, 43, 47, 50, 52, 55, 56, 58, 60, 64–68]	“we started one patient on insulin in the community which is fantastic, saved so much hassle for a demented man not to have to go into hospital” [31] “The odd time you get people in who are, live on the streets, you know, I’d prescribe for them, and you can get those things over-the-counter because they haven’t got the money and they get free prescriptions” [52]	Long-term conditions such as diabetes. Complex patients such as those with comorbidities. Minor ailments. Patients with social needs for example drug users.
	3.3.4.1. Setting [31, 36, 37, 43–45, 47, 49–52, 54, 56–58, 63, 64, 66, 68]	“A major benefit of seeing the patient in their home, in a setting where it's to their best convenience” [63] “My main dealings are treating people with acute respiratory problems. Their medicines’ (ran) out, or they’re becoming ill with complications. That’s mainly an out of hours setting. It is a benefit for them to walk in to the walk-in centre. At least they’re getting care somewhere.” [37]	Primary and secondary care, including cross sector working, ranging from home based care to specialist clinic.

### Non-medical prescriber themes

Factors affecting the NMP were subdivided into those arising from the attitude of the NMP and those derived from their practice (See Table 3). Prescribing enabled the professional to practice autonomously [21, 28, 31, 37, 42, 46, 65], enhancing job satisfaction [31, 37, 42, 46, 47, 49, 65], and supporting professional development [27, 33, 47, 50]. Some practitioners, however, expressed anxiety [29, 37] and cautiousness [27, 48, 52, 65]. Practitioners indicated that their area of competency enabled them to prescribe confidently [44, 48, 52, 65–67], and to resist pressure to prescribe outside this area [34, 44, 52, 65–67]. Roles were enhanced through including prescribing [27, 33, 35, 37, 42, 44, 58, 63, 67].

### Human factor themes

Human factors described the impact that NMPs had on their patients, colleagues, and managers, and the impact that these people had on the NMP themselves. Medical staff that had been involved in the training of NMPs [39, 54, 63] were more supportive than those who were unaware of the training involved [39, 43]. This was regardless of seniority [55, 66]; junior medical staff were less likely to be supportive [39]. Managers were instrumental in developing and supporting the NMP role [27, 36, 41, 43]. Lack of support, flexibility or understanding by managers hindered the implementation and development of non-medical prescribing [27, 29, 31, 32, 37, 46, 52, 54, 61, 66, 67]. NMPs gained support from colleagues, describing enhanced team working [27, 32, 34, 35, 41, 43, 47–49, 55, 57, 65–67], and were perceived as supportive experts and leaders [27, 32, 43, 47, 67]. However, NMPs encountered opposition from some colleagues [21, 27, 31, 32, 38, 43, 44, 47, 52, 62, 66].

### Organisational aspect themes

Organisational aspects encompassed a range of themes covering administration, development and service delivery. Administration comprised three subthemes: formulary, policy, and remuneration. A formulary could be self-imposed [27, 31, 32, 48, 52], or organisation derived [27, 29, 31, 32, 36, 62], and while they could be empowering [31, 36, 52, 66], they could be restrictive [27, 29, 32, 36, 48, 52, 62]. Local policies could be supportive [27, 47, 66], restrictive [27, 31, 43, 66], or missing [62]. Remuneration was considered to be non-commensurate with skills [27, 43, 46, 50, 54, 60, 62]. Development covered both training, including selection for course, as well as post course support. Course facilitators included appropriate selection of candidates [35, 39, 41, 45, 47, 50], awareness of course commitments and requirements [48], and support from medical mentors [43, 63], and managers [39, 41, 45]. Post course support included the provision or facilitation of professional development courses [27, 36, 41, 47, 48, 67], mentoring [27, 41, 48, 50], and clinical supervision [27, 36, 66]. Absence of such support hindered NMP development [27, 33, 35–37, 43, 46, 48, 52, 62, 63, 66, 67]. Infrastructure covered several issues, each with the potential to support or hinder, including access to: patient records [27, 37, 43, 46, 49, 51, 52, 54, 63, 64], information technology [27, 31, 36, 38, 43, 48], prescriptions [27, 31, 32, 37, 38, 43, 62, 67], and facilities [43, 49]. NMPs spent more time with patients [35, 37, 39, 47, 49, 52, 55, 56, 63, 68], and were considered to provide a responsive, efficient, and convenient service [27, 29, 33, 35–37, 40, 44, 47–49, 59, 65, 68]. Doctors’ time was released by NMPs activity [29, 36, 43, 51, 63, 67], but time constraints and workload could hinder the NMP service [29, 34, 35, 44, 46, 49, 52, 63]. Some services were now reliant on NMPs [36, 37] and had issues when cover was absent [36]. The settings and patient groups where non-medical prescribing is utilised were diverse. Examples were given of utilising non-medical prescribing to treat patients who may find accessing healthcare difficult such as frail and housebound patients [37, 52, 63], the homeless [52], and drug users [43, 58]. Non-medical prescribing was also utilised in more conventional healthcare settings such as specialist clinics (for example, dermatology [36, 43], anti-coagulation [56], and cardiovascular [43]), minor illness clinics [31, 36, 37, 44, 50], and out-of-hours services [36, 37, 52].

During analysis, it became apparent that many factors were not present in isolation but were interdependent. Frequently, the interdependence was between a member of staff, the NMP, an organisational aspect such as policy, and how this impacted on the NMP’s confidence and ability to prescribe. Examples include a situation whereby a supportive GP had given an NMP confidence to develop her competence area and expand her personal prescribing formulary [27], and identification by NMP leads that an NMP role was more likely to flourish when linked to a strategic vision and a well-defined area of practice [41]. Other interdependencies were within organisational aspects, such as the increased time required when the NMP was unable to easily access the patient’s notes [37], or when the non-medical prescribing policy specifically supported access to continuing training [28].

## Discussion

This is the first systematic review to investigate and synthesise the qualitative and mixed methods literature regarding barriers and facilitators to, and use of, independent non-medical prescribing. Three overarching themes, each containing subthemes, were identified; the NMP, human factors and organisational aspects. The themes and subthemes could all impact on successful implementation of non-medical prescribing, and could be interdependent.

The NMP theme describes three aspects; one is intrinsic to the person (attitude), one derives from their role, and the final one may be personally or externally derived. The later subtheme ‘Area of competence’ was one of the four most highly mentioned aspects found during analysis, highlighting its importance. This is supported by the ‘Competency framework for all prescribers’ [8] and the NMC ‘Standards of proficiency for nurse and midwife prescribers’ [9], which state that practitioners should only prescribe within their scope of practice (in contrast with the traditional medical model). There are implications if the NMP changes role, or in planned service expansion, as further training and support in these new areas would be required. Closely defined areas of competence could hamper full utilisation of non-medical prescribing, particularly in patients with co-morbidities.

The second theme ‘human factors’ describes the complex interrelation between the NMP, their managers, peers, the medical professions they work with, and their patients. This theme included the most frequently mentioned subtheme ‘Medical professionals’, identified in 32 papers. It is notable that, in contrast with the review by Cooper et al, medical professionals generally accepted the NMP role [16]. Reasons for acceptance may be because non-medical prescribing has become established practice but also because NMPs have made deliberate efforts to gain trust. There was an appreciation that the NMP role permitted medical professionals to concentrate on patients where their expertise was necessary. Changes in managerial personnel could adversely impact on non-medical prescribing, particularly where systems and processes were not embedded into practice. This review found that patients’ views of non-medical prescribing were mixed, with many patients appreciating the time taken and holistic approach of the NMP, whereas others expressed concerns. A lack of public understanding of non-medical prescribing remains, even with patients treated by NMPs. Cooper et al noted that very little research was identified investigating the views of patients about non-medical prescribing [16]. This review identified one paper investigating public perception of non-medical prescribing [51] and eight papers that included the views of patients [27, 28, 40, 49, 56, 59, 61, 68]; however, one of these only included quantitative ‘rating’ data from patients [40]. Research into patients’ opinions of non-medical prescribing warrants further investigation.

The final theme covers the organisational aspects that support and enable an NMP to practice. It contains two of the four most frequently mentioned subthemes, ‘impact on time’ and ‘service’. In comparison to other subthemes, these two were frequently interdependent on each other, with both highlighting the perceived improvement to patient care by providing a streamlined, holistic, and convenient service. Funding pressures may make this aspect of the service, appreciated by patients, difficult to sustain. This review identified that contingency and succession planning should be considered during service development.

This review’s strength lies in its rigorous methodology and breadth of search strategy. This compares with the previous investigations, which were limited in scope and rigour [14, 16]. The predetermined stringent protocol, registered with PROSPERO, and the use of two independent reviewers are recognised strategies to reduce potential bias associated with paper selection [20, 71]. Limitations included the inconsistent definitions used to describe NMPs, which became apparent during the literature search. The terminology would have been appropriate when those studies were conducted, but the meaning changed as prescribing rights evolved (see Table 1). Every effort was made to limit the included studies to those investigating full independent non-medical prescribing. The nursing profession dominated the included studies, with limited representation from pharmacist prescribers (mentioned in 11 papers [27, 33, 43, 47, 49, 52–55, 57, 61]) and none from other non-medical prescribing professions. This reflects the relative numbers of the different professions [15, 72] and the numbers of qualified prescribers [12]. However, the numbers of AHPS are likely to have increased recently following legislation changes and that could be considered a limitation. Research into non-medical prescribing by the other professions is needed to identify if they experience the same barriers and facilitators.

The themes and subthemes identified in this review influence the implementation and development of non-medical prescribing; each could act as a barrier or facilitator depending on circumstances. Where there was a lack of understanding of the non-medical prescribing role, or lack of trust in the non-medical prescriber, then the factors were more inclined to be barriers. For example, medical professionals were less likely to support non-medical prescribing where there was a lack of clarity about who took responsibility for the prescribing practice [35, 39, 50]. Facilitation of NMP occurred when medical professionals trusted the NMP, for example enabling access to patient records [37]. As a consequence of budgetary constraints, factors may become barriers, such as the use of restrictive formularies as a cost saving measure [37, 52, 64]. Additionally, this review has identified that these themes and subthemes do not stand in isolation but are interdependent on each other. Each of these aspects should be considered when developing a non-medical prescribing service, and could be utilised as a model for developing a non-medical prescribing strategy framework. This review will also inform those currently managing or running a service, enabling service optimisation. Failure to address all these aspects may mean that the full benefit of an NMP service will not be realised.

## Supporting information

S1 Appendix PRISMA checklist. (DOC)

S2 Appendix ENTREQ checklist. (DOCX)

S3 Appendix Medline (Ovid) search strategy. (DOCX)

S1 Protocol PROSPERO record. (PDF)

S1 Table QATSDD scores for each paper. (DOCX)

S2 Table Themes identified in each paper. (DOCX)
