# Supplementary material for: Facilitators and barriers to non-medical prescribing – A systematic review and thematic synthesis
Source: PLoS One. 2018 Apr 30;13(4):e0196471. doi: 10.1371/journal.pone.0196471 (PMC5927440; doi:10.1371/journal.pone.0196471)
Supplement: S1 Table — (DOCX) [file pone.0196471.s005.docx]

# S1 Table. QATSDD scores for each paper

|  | **Paper Reference Number** | | | | | | | | | | | | | | | | | | | | | | | | | | | | | | | | | | | | | | | | | |
| --- | --- | --- | --- | --- | --- | --- | --- | --- | --- | --- | --- | --- | --- | --- | --- | --- | --- | --- | --- | --- | --- | --- | --- | --- | --- | --- | --- | --- | --- | --- | --- | --- | --- | --- | --- | --- | --- | --- | --- | --- | --- | --- |
| **Assessment Criteria [1]** | **2** | **3** | **4** | **5** | **6** | **7** | **8** | **9** | **10** | **11** | **12** | **13** | **14** | **15** | **16** | **17** | **18** | **19** | **20** | **21** | **22** | **23** | **24** | **25** | **26** | **27** | **28** | **29** | **30** | **31** | **32** | **33** | **34** | **35** | **36** | **37** | **38** | **39** | **40** | **41** | **42** | **43** |
| Explicit theoretical framework | 3 | 2 | 0 | 0 | 3 | 0 | 1 | 1 | 1 | 1 | 1 | 1 | 2 | 1 | 1 | 1 | 3 | 0 | 1 | 3 | 0 | 1 | 0 | 0 | 1 | 3 | 0 | 0 | 2 | 0 | 0 | 0 | 1 | 3 | 3 | 1 | 2 | 1 | 0 | 2 | 1 | 1 |
| Statement of aims/objectives in main body of report | 1 | 3 | 3 | 2 | 3 | 3 | 2 | 3 | 3 | 3 | 3 | 3 | 3 | 3 | 2 | 2 | 3 | 3 | 2 | 3 | 1 | 3 | 0 | 3 | 3 | 3 | 2 | 3 | 2 | 2 | 2 | 3 | 3 | 1 | 3 | 3 | 3 | 3 | 3 | 3 | 3 | 3 |
| Clear description of research setting | 3 | 2 | 2 | 2 | 3 | 3 | 3 | 3 | 2 | 1 | 3 | 3 | 3 | 3 | 3 | 3 | 3 | 3 | 3 | 1 | 3 | 2 | 1 | 2 | 3 | 3 | 2 | 2 | 2 | 2 | 2 | 1 | 3 | 2 | 3 | 2 | 2 | 2 | 2 | 2 | 2 | 3 |
| Evidence of sample size considered in terms of analysis | 2 | 1 | 1 | 0 | 3 | 3 | 0 | 1 | 2 | 1 | 0 | 3 | 1 | 3 | 1 | 3 | 3 | 0 | 1 | 0 | 2 | 0 | 0 | 1 | 0 | 3 | 3 | 2 | 2 | 1 | 2 | 1 | 1 | 1 | 2 | 1 | 0 | 2 | 2 | 3 | 1 | 0 |
| Representative sample of target group of a reasonable size | 3 | 1 | 3 | 1 | 3 | 3 | 2 | 2 | 2 | 2 | 3 | 3 | 3 | 3 | 3 | 3 | 3 | 1 | 3 | 0 | 3 | 2 | 2 | 3 | 2 | 3 | 3 | 3 | 3 | 2 | 2 | 1 | 2 | 2 | 2 | 2 | 2 | 3 | 3 | 2 | 2 | 3 |
| Description of procedure for data collection | 3 | 1 | 3 | 1 | 3 | 3 | 2 | 2 | 2 | 2 | 2 | 3 | 3 | 3 | 3 | 2 | 3 | 2 | 3 | 2 | 2 | 2 | 3 | 1 | 1 | 3 | 3 | 2 | 3 | 3 | 1 | 1 | 2 | 3 | 1 | 3 | 3 | 2 | 2 | 2 | 2 | 3 |
| Rationale for choice of data collection tool(s) | 3 | 1 | 3 | 0 | 3 | 0 | 0 | 0 | 0 | 0 | 0 | 2 | 3 | 2 | 2 | 1 | 3 | 0 | 2 | 3 | 3 | 1 | 0 | 0 | 0 | 3 | 2 | 0 | 0 | 3 | 0 | 0 | 2 | 2 | 0 | 3 | 0 | 0 | 0 | 3 | 0 | 0 |
| Detailed recruitment data | 3 | 2 | 3 | 0 | 3 | 2 | 1 | 3 | 2 | 1 | 3 | 3 | 3 | 3 | 3 | 2 | 3 | 2 | 3 | 1 | 3 | 3 | 2 | 2 | 0 | 3 | 3 | 3 | 3 | 3 | 2 | 2 | 3 | 3 | 2 | 3 | 2 | 1 | 1 | 2 | 2 | 3 |
| Statistical assessment of reliability & validity of measurement tool(s) (Quantitative only) | n/a | n/a | n/a | n/a | n/a | n/a | n/a | n/a | n/a | n/a | n/a | 0 | n/a | 0 | 0 | n/a | 3 | n/a | 0 | n/a | n/a | n/a | n/a | 0 | n/a | n/a | n/a | 1 | n/a | n/a | 0 | n/a | 0 | n/a | n/a | 1 | n/a | n/a | n/a | n/a | n/a | n/a |
| Fit between stated research question & method of data collection (Quantitative) | n/a | n/a | n/a | n/a | n/a | n/a | n/a | n/a | n/a | n/a | n/a | 0 | n/a | 0 | 0 | n/a | 3 | n/a | 0 | n/a | n/a | n/a | n/a | 0 | n/a | n/a | n/a | 0 | n/a | n/a | 0 | n/a | 0 | n/a | n/a | 0 | n/a | n/a | n/a | n/a | n/a | n/a |
| Fit between stated research question & format & content of data collection tool (Qualitative) | 3 | 0 | 0 | 0 | 3 | 3 | 0 | 0 | 0 | 0 | 0 | 0 | 0 | 0 | 0 | 0 | 3 | 0 | 0 | 0 | 0 | 0 | 0 | 0 | 0 | 3 | 0 | 0 | 0 | 0 | 0 | 0 | 0 | 0 | 0 | 0 | 0 | 0 | 0 | 0 | 0 | 0 |
| Fit between research question & method of analysis | 3 | 3 | 0 | 0 | 3 | 3 | 2 | 3 | 0 | 0 | 3 | 3 | 3 | 3 | 3 | 3 | 3 | 3 | 2 | 3 | 0 | 3 | 2 | 3 | 3 | 3 | 3 | 0 | 3 | 3 | 0 | 0 | 0 | 3 | 3 | 3 | 3 | 3 | 3 | 3 | 3 | 3 |
| Good justification for analytical method selected | 3 | 0 | 1 | 0 | 3 | 1 | 0 | 0 | 1 | 3 | 1 | 0 | 1 | 0 | 0 | 0 | 3 | 0 | 0 | 3 | 0 | 2 | 0 | 0 | 2 | 3 | 1 | 1 | 1 | 2 | 0 | 0 | 0 | 3 | 1 | 3 | 2 | 2 | 1 | 1 | 0 | 0 |
| Assessment of reliability of analytical process (Qualitative only) | 3 | 0 | 0 | 0 | 2 | 0 | 0 | 3 | 3 | 3 | 3 | 0 | 3 | 3 | 2 | 3 | 2 | 0 | 0 | 0 | 0 | 0 | 1 | 0 | 0 | 3 | 3 | 0 | 3 | 2 | 0 | 0 | 0 | 3 | 2 | 3 | 2 | 3 | 3 | 3 | 3 | 3 |
| Evidence of user involvement in design | 2 | 1 | 0 | 0 | 3 | 1 | 0 | 0 | 0 | 0 | 1 | 1 | 0 | 0 | 1 | 0 | 3 | 0 | 0 | 0 | 2 | 0 | 1 | 2 | 2 | 1 | 1 | 2 | 0 | 0 | 0 | 0 | 0 | 1 | 1 | 3 | 2 | 0 | 0 | 0 | 0 | 0 |
| Strengths & limitations critically discussed | 3 | 2 | 0 | 0 | 0 | 0 | 3 | 2 | 2 | 2 | 3 | 2 | 2 | 1 | 1 | 1 | 3 | 2 | 2 | 1 | 1 | 2 | 2 | 0 | 1 | 3 | 3 | 1 | 1 | 3 | 0 | 0 | 0 | 3 | 2 | 3 | 1 | 2 | 2 | 2 | 2 | 2 |
| **Total** | 38 | 19 | 19 | 6 | 38 | 25 | 16 | 23 | 20 | 19 | 26 | 27 | 30 | 28 | 25 | 24 | 47 | 16 | 22 | 20 | 20 | 21 | 14 | 17 | 18 | 40 | 29 | 20 | 25 | 26 | 11 | 9 | 17 | 30 | 25 | 34 | 24 | 24 | 22 | 28 | 21 | 24 |
| Maximum score possible | 42 | 42 | 42 | 42 | 42 | 42 | 42 | 42 | 42 | 42 | 42 | 48 | 42 | 48 | 48 | 42 | 48 | 42 | 48 | 42 | 42 | 42 | 42 | 48 | 42 | 42 | 42 | 48 | 42 | 42 | 48 | 42 | 48 | 42 | 42 | 48 | 42 | 42 | 42 | 42 | 42 | 42 |
| Score as Percentage (%) | 90% | 45% | 45% | 14% | 90% | 60% | 38% | 55% | 48% | 45% | 62% | 56% | 71% | 58% | 52% | 57% | 98% | 38% | 46% | 48% | 48% | 50% | 33% | 35% | 43% | 95% | 69% | 42% | 60% | 62% | 23% | 21% | 35% | 71% | 60% | 71% | 57% | 57% | 52% | 67% | 50% | 57% |

n/a = not applicable

## References

1. Sirriyeh R, Lawton R, Gardner P, Armitage G. Reviewing studies with diverse designs: the development and evaluation of a new tool. J Eval Clin Pract. 2012;18(4):746-52. doi: 10.1111/j.1365-2753.2011.01662.x.
2. Adigwe OP. Non-medical prescribing in chronic non-malignant pain [PhD]. Leeds: University of Leeds; 2012.
3. Armstrong A. Staff and patient views on nurse prescribing in the urgent-care setting. Nurse Prescribing. 2015;13(12):614-9. doi: 10.12968/npre.2015.13.12.614.
4. Bennett J, Jones M. Nurse prescribing in HIV: opportunities and threats. HIV Nursing. 2008;8(4):12-6.
5. Bewley T. Preparation for non medical prescribing: a review. Paediatr Nurs. 2007;19(5):23-6.
6. Bowskill D. The integration of nurse prescribing: case studies in primary and secondary care [DHSci]. Nottingham: University of Nottingham; 2009.
7. Bowskill D, Timmons S, James V. How do nurse prescribers integrate prescribing in practice: case studies in primary and secondary care. J Clin Nurs. 2013;22(13-14):2077-86. doi: 10.1111/j.1365-2702.2012.04338.x.
8. Brodie L, Donaldson J, Watt S. Non-medical prescribers and benzodiazepines: a qualitative study. Nurse Prescribing. 2014;12(7):353-9. doi: 10.12968/npre.2014.12.7.353.
9. Carey N, Stenner K, Courtenay M. Adopting the prescribing role in practice: exploring nurses' views in a specialist children's hospital. Paediatr Nurs. 2009;21(9):25-9. doi: 10.7748/paed2009.11.21.9.25.c7357.
10. Carey N, Stenner K, Courtenay M. Views on implementing nurse prescribing in a specialist children's hospital. Nurse Prescribing. 2009;7(5):205-10. doi: 10.12968/npre.2009.7.5.42356.
11. Carey N, Stenner K, Courtenay M. Stakeholder views on the impact of nurse prescribing on dermatology services. J Clin Nurs. 2010;19(3-4):498-506. doi: 10.1111/j.1365-2702.2009.02874.x.
12. Carey N, Stenner K, Courtenay M. An exploration of how nurse prescribing is being used for patients with respiratory conditions across the east of England. BMC Health Serv Res. 2014;14:13. doi: 10.1186/1472-6963-14-27.
13. Courtenay M, Carey N. Nurse independent prescribing and nurse supplementary prescribing practice: national survey. J Adv Nurs. 2008;61(3):291-9. doi: 10.1111/j.1365-2648.2007.04512.x.
14. Courtenay M, Carey N. Nurse prescribing by children's nurses: views of doctors and clinical leads in one specialist children's hospital. J Clin Nurs. 2009;18(18):2668-75. doi: 10.1111/j.1365-2702.2009.02799.x.
15. Courtenay M, Carey N, Stenner K. Nurse prescriber-patient consultations: a case study in dermatology. J Adv Nurs. 2009;65(6):1207-17. doi: 10.1111/j.1365-2648.2009.04974.x.
16. Courtenay M, Carey N, Stenner K. Non medical prescribing leads views on their role and the implementation of non medical prescribing from a multi-organisational perspective. BMC Health Serv Res. 2011;11:142. doi: 10.1186/1472-6963-11-142.
17. Cousins R, Donnell C. Nurse prescribing in general practice: a qualitative study of job satisfaction and work-related stress. Fam Pract. 2012;29(2):223-7. doi: 10.1093/fampra/cmr077.
18. Dapar MP. An investigation of the structures and processes of pharmacist prescribing in Great Britain: a mixed methods approach [PhD]. Aberdeen: Robert Gordon University; 2012.
19. Daughtry J, Hayter M. A qualitative study of practice nurses' prescribing experiences. Practice Nursing. 2010;21(6):310-4. doi: 10.12968/pnur.2010.21.6.48329.
20. Dobel-Ober D, Brimblecombe N, Bradley E. Nurse prescribing in mental health: national survey. J Psychiatr Ment Health Nurs. 2010;17(6):487-93. doi: 10.1111/j.1365-2850.2009.01541.x.
21. Downer F, Shepherd CK. District nurses prescribing as nurse independent prescribers. Br J Community Nurs. 2010;15(7):348-52. doi: 10.12968/bjcn.2010.15.7.48774.
22. Green B, Courtney H. Evaluating the investment: a survey of non-medical prescribing. Mental Health Practice. 2008;12(1):28-32.
23. Herklots A, Baileff A, Latter S. Community matrons' experience as independent prescribers. Br J Community Nurs. 2015;20(5):217-23. doi: 10.12968/bjcn.2015.20.5.217.
24. Hill DR, Conroy S, Brown RC, Burt GA, Campbell D. Stakeholder views on pharmacist prescribing in addiction services in NHS Lanarkshire. J Subst Use. 2014;19(1-2):56-67. doi: 10.3109/14659891.2012.734540.
25. Kelly A, Neale J, Rollings R. Barriers to extended nurse prescribing among practice nurses. Community Pract. 2010;83(1):21-4.
26. Maclure K, George J, Diack L, Bond C, Cunningham S, Stewart D. Views of the Scottish general public on non-medical prescribing. Int J Clin Pharm. 2013;35(5):704-10. doi: 10.1007/s11096-013-9792-x.
27. Maddox C. Influences on non-medical prescribing: nurse and pharmacist prescribers in primary and community care [PhD]. Manchester: University of Manchester; 2011.
28. Maddox C, Halsall D, Hall J, Tully MP. Factors influencing nurse and pharmacist willingness to take or not take responsibility for non-medical prescribing. Res Social Adm Pharm. 2016;12(1):41-55. doi: <http://dx.doi.org/10.1016/j.sapharm.2015.04.001>.
29. McCann L, Haughey S, Parsons C, Lloyd F, Crealey G, Gormley GJ, et al. Pharmacist prescribing in Northern Ireland: a quantitative assessment. Int J Clin Pharm. 2011;33(5):824-31. doi: <http://dx.doi.org/10.1007/s11096-011-9545-7>.
30. McCann L, Lloyd F, Parsons C, Gormley G, Haughey S, Crealey G, et al. "They come with multiple morbidities": A qualitative assessment of pharmacist prescribing. J Interprof Care. 2012;26(2):127-33. doi: 10.3109/13561820.2011.642425.
31. McCann LM, Haughey SL, Parsons C, Lloyd F, Crealey G, Gormley GJ, et al. A patient perspective of pharmacist prescribing: 'crossing the specialisms-crossing the illnesses'. Health Expect. 2015;18(1):58-68. doi: 10.1111/hex.12008.
32. Mulholland PJ. Pharmacists as non-medical prescribers: what role can they play? The experience in a neonatal intensive care unit. Eur J Hosp Pharm-Sci Pract. 2014;21(6):335-8. doi: 10.1136/ejhpharm-2013-000401.
33. Mundt-Leach R. Non-medical prescribing by specialist addictions nurses. Mental Health Practice. 2012;16(3):28-31.
34. Oldknow H, Bottomley J, Lawton M. Independent nurse prescribing for older people's mental health. Nurse Prescribing. 2010;8(2):66-9. doi: 10.12968/npre.2010.8.2.46527.
35. Oldknow H, Gillibrand W. Non-prescribing, non-medical prescribers: a qualitative exploratory enquiry - preliminary findings. Mental Health Nursing. 2013;33(4):10-3.
36. Ross JD. Mental health nurse prescribing: the emerging impact. J Psychiatr Ment Health Nurs. 2015;22(7):529-42. doi: 10.1111/jpm.12207.
37. Ross JD, Kettles AM. Mental health nurse independent prescribing: what are nurse prescribers' views of the barriers to implementation? J Psychiatr Ment Health Nurs. 2012;19(10):916-32. doi: 10.1111/j.1365-2850.2011.01872.x.
38. Shannon E, Spence W. The attitudes and views of GPs and physicians to prescribing by heart failure nurse specialists. British Journal of Cardiac Nursing. 2011;6(9):450-5. doi: 10.12968/bjca.2011.6.9.450.
39. Stenner K, Courtenay M. A qualitative study on the impact of legislation on prescribing of controlled drugs by nurses. Nurse Prescribing. 2007;5(6):257-61. doi: 10.12968/npre.2007.5.6.24292.
40. Stenner K, Courtenay M. Benefits of nurse prescribing for patients in pain: nurses' views. J Adv Nurs. 2008;63(1):27-35. doi: 10.1111/j.1365-2648.2008.04644.x.
41. Stenner K, Courtenay M. The role of inter-professional relationships and support for nurse prescribing in acute and chronic pain. J Adv Nurs. 2008;63(3):276-83. doi: 10.1111/j.1365-2648.2008.04707.x.
42. Stenner K, Carey N, Courtenay M. Implementing nurse prescribing: a case study in diabetes. J Adv Nurs. 2010;66(3):522-31. doi: 10.1111/j.1365-2648.2009.05212.x.
43. Stenner KL, Courtenay M, Carey N. Consultations between nurse prescribers and patients with diabetes in primary care: A qualitative study of patient views. Int J Nurs Stud. 2011;48(1):37-46. doi: 10.1016/j.ijnurstu.2010.06.006.
