# Supplementary material for: Facilitators and barriers to non-medical prescribing – A systematic review and thematic synthesis
Source: PLoS One. 2018 Apr 30;13(4):e0196471. doi: 10.1371/journal.pone.0196471 (PMC5927440; doi:10.1371/journal.pone.0196471)
Supplement: S2 Table — (DOCX) [file pone.0196471.s006.docx]

# S2 Table: Themes identified in each paper

| **Theme** | **Paper Reference Number** |
| --- | --- |
| 1. **Non-medical prescriber** | |
| - 1. Attitude | 1,2,3,5,6,7,8,9,11,14,16,18,20,21,22,23,24,26,27,35,39,40,41, |
| - 1. Practice | |
| - - 1. Area of competence | 1,5,6,7,8,9,11,12,14,16,17,18,22,23,24,25,26,27,29,30,31,32,39,40,41,42, |
| - - 1. Role | 1,2,5,6,7,8,9,10,11,16,17,18,20,21,24,26,27,29,30,32,36,37,39,40,41, |
| 1. **Human factors** | |
| - 1. Patients | 1,2,3,17,21,23,25,30,33,35,37,41,42, |
| - 1. Staff | |
| - - 1. Managers | 1,3,5,6,7,9,10,11,15,17,19,20,24,26,28,34,36,40,41, |
| - - 1. Medical Professionals | 1,2,3,5,6,7,8,9,10,11,12,13,17,18,20,21,22,23,24,26,27,28,29,31,33,34,35,36,37,39,40,41, |
| - - 1. Peers | 1,2,5,6,8,9,10,12,15,17,18,20,21,22,23,26,27,29,31,32,36,39,40,41, |
| 1. **Organisational aspects** | |
| - 1. Administration | |
| - - 1. Formulary | 1,3,5,6,7,10,11,17,21,22,25,26,36,38,40, |
| - - 1. Policy | 1,2,5,10,17,21,36,40, |
| - - 1. Remuneration | 1,11,17,20,24,28,34,36, |
| - 1. Development | |
| - - 1. Post Course support | 1,2,7,9,10,11,13,15,17,20,21,22,24,25,26,27,29,31,36,37,39,40,41, |
| - - 1. Training | 1,2,4,7,9,13,15,17,19,20,21,22,24,26,27,30,31,36,37,41, |
| - 1. Service delivery | |
| - - 1. Impact on time | 1,2,3,7,8,9,10,11,13,14,17,18,20,21,22,23,25,26,29,30,31,32,33,35,37,39,41,42, |
| - - 1. Infrastructure | 1,5,6,7,8,10,11,12,17,20,21,22,23,25,26,28,31,36,37,38,41, |
| - - 1. Service | 1,2,3,7,8,9,10,11,13,14,15,16,17,18,21,22,23,26,27,29,30,31,32,33,35,37,39,42, |
| - - 1. Use in practice | |
| - - - 1. Patients | 3,5,6,7,8,9,10,11,13,17,21,24,26,29,30,32,34,38,39,40,41,42, |
| - - - 1. Setting | 5,10,11,17,18,19,21,23,24,25,26,28,30,31,32,37,38,40,42, |

## References

1. Adigwe OP. Non-medical prescribing in chronic non-malignant pain [PhD]. Leeds: University of Leeds; 2012.
2. Armstrong A. Staff and patient views on nurse prescribing in the urgent-care setting. Nurse Prescribing. 2015;13(12):614-9. doi: 10.12968/npre.2015.13.12.614.
3. Bennett J, Jones M. Nurse prescribing in HIV: opportunities and threats. HIV Nursing. 2008;8(4):12-6.
4. Bewley T. Preparation for non medical prescribing: a review. Paediatr Nurs. 2007;19(5):23-6.
5. Bowskill D. The integration of nurse prescribing: case studies in primary and secondary care [DHSci]. Nottingham: University of Nottingham; 2009.
6. Bowskill D, Timmons S, James V. How do nurse prescribers integrate prescribing in practice: case studies in primary and secondary care. J Clin Nurs. 2013;22(13-14):2077-86. doi: 10.1111/j.1365-2702.2012.04338.x.
7. Brodie L, Donaldson J, Watt S. Non-medical prescribers and benzodiazepines: a qualitative study. Nurse Prescribing. 2014;12(7):353-9. doi: 10.12968/npre.2014.12.7.353.
8. Carey N, Stenner K, Courtenay M. Adopting the prescribing role in practice: exploring nurses' views in a specialist children's hospital. Paediatr Nurs. 2009;21(9):25-9. doi: 10.7748/paed2009.11.21.9.25.c7357.
9. Carey N, Stenner K, Courtenay M. Views on implementing nurse prescribing in a specialist children's hospital. Nurse Prescribing. 2009;7(5):205-10. doi: 10.12968/npre.2009.7.5.42356.
10. Carey N, Stenner K, Courtenay M. Stakeholder views on the impact of nurse prescribing on dermatology services. J Clin Nurs. 2010;19(3-4):498-506. doi: 10.1111/j.1365-2702.2009.02874.x.
11. Carey N, Stenner K, Courtenay M. An exploration of how nurse prescribing is being used for patients with respiratory conditions across the east of England. BMC Health Serv Res. 2014;14:13. doi: 10.1186/1472-6963-14-27.
12. Courtenay M, Carey N. Nurse independent prescribing and nurse supplementary prescribing practice: national survey. J Adv Nurs. 2008;61(3):291-9. doi: 10.1111/j.1365-2648.2007.04512.x.
13. Courtenay M, Carey N. Nurse prescribing by children's nurses: views of doctors and clinical leads in one specialist children's hospital. J Clin Nurs. 2009;18(18):2668-75. doi: 10.1111/j.1365-2702.2009.02799.x.
14. Courtenay M, Carey N, Stenner K. Nurse prescriber-patient consultations: a case study in dermatology. J Adv Nurs. 2009;65(6):1207-17. doi: 10.1111/j.1365-2648.2009.04974.x.
15. Courtenay M, Carey N, Stenner K. Non medical prescribing leads views on their role and the implementation of non medical prescribing from a multi-organisational perspective. BMC Health Serv Res. 2011;11:142. doi: 10.1186/1472-6963-11-142.
16. Cousins R, Donnell C. Nurse prescribing in general practice: a qualitative study of job satisfaction and work-related stress. Fam Pract. 2012;29(2):223-7. doi: 10.1093/fampra/cmr077.
17. Dapar MP. An investigation of the structures and processes of pharmacist prescribing in Great Britain: a mixed methods approach [PhD]. Aberdeen: Robert Gordon University; 2012.
18. Daughtry J, Hayter M. A qualitative study of practice nurses' prescribing experiences. Practice Nursing. 2010;21(6):310-4. doi: 10.12968/pnur.2010.21.6.48329.
19. Dobel-Ober D, Brimblecombe N, Bradley E. Nurse prescribing in mental health: national survey. J Psychiatr Ment Health Nurs. 2010;17(6):487-93. doi: 10.1111/j.1365-2850.2009.01541.x.
20. Downer F, Shepherd CK. District nurses prescribing as nurse independent prescribers. Br J Community Nurs. 2010;15(7):348-52. doi: 10.12968/bjcn.2010.15.7.48774.
21. Green B, Courtney H. Evaluating the investment: a survey of non-medical prescribing. Mental Health Practice. 2008;12(1):28-32.
22. Herklots A, Baileff A, Latter S. Community matrons' experience as independent prescribers. Br J Community Nurs. 2015;20(5):217-23. doi: 10.12968/bjcn.2015.20.5.217.
23. Hill DR, Conroy S, Brown RC, Burt GA, Campbell D. Stakeholder views on pharmacist prescribing in addiction services in NHS Lanarkshire. J Subst Use. 2014;19(1-2):56-67. doi: 10.3109/14659891.2012.734540.
24. Kelly A, Neale J, Rollings R. Barriers to extended nurse prescribing among practice nurses. Community Pract. 2010;83(1):21-4.
25. Maclure K, George J, Diack L, Bond C, Cunningham S, Stewart D. Views of the Scottish general public on non-medical prescribing. Int J Clin Pharm. 2013;35(5):704-10. doi: 10.1007/s11096-013-9792-x.
26. Maddox C. Influences on non-medical prescribing: nurse and pharmacist prescribers in primary and community care [PhD]. Manchester: University of Manchester; 2011.
27. Maddox C, Halsall D, Hall J, Tully MP. Factors influencing nurse and pharmacist willingness to take or not take responsibility for non-medical prescribing. Res Social Adm Pharm. 2016;12(1):41-55. doi: <http://dx.doi.org/10.1016/j.sapharm.2015.04.001>.
28. McCann L, Haughey S, Parsons C, Lloyd F, Crealey G, Gormley GJ, et al. Pharmacist prescribing in Northern Ireland: a quantitative assessment. Int J Clin Pharm. 2011;33(5):824-31. doi: <http://dx.doi.org/10.1007/s11096-011-9545-7>.
29. McCann L, Lloyd F, Parsons C, Gormley G, Haughey S, Crealey G, et al. "They come with multiple morbidities": A qualitative assessment of pharmacist prescribing. J Interprof Care. 2012;26(2):127-33. doi: 10.3109/13561820.2011.642425.
30. McCann LM, Haughey SL, Parsons C, Lloyd F, Crealey G, Gormley GJ, et al. A patient perspective of pharmacist prescribing: 'crossing the specialisms-crossing the illnesses'. Health Expect. 2015;18(1):58-68. doi: 10.1111/hex.12008.
31. Mulholland PJ. Pharmacists as non-medical prescribers: what role can they play? The experience in a neonatal intensive care unit. Eur J Hosp Pharm-Sci Pract. 2014;21(6):335-8. doi: 10.1136/ejhpharm-2013-000401.
32. Mundt-Leach R. Non-medical prescribing by specialist addictions nurses. Mental Health Practice. 2012;16(3):28-31.
33. Oldknow H, Bottomley J, Lawton M. Independent nurse prescribing for older people's mental health. Nurse Prescribing. 2010;8(2):66-9. doi: 10.12968/npre.2010.8.2.46527.
34. Oldknow H, Gillibrand W. Non-prescribing, non-medical prescribers: a qualitative exploratory enquiry - preliminary findings. Mental Health Nursing. 2013;33(4):10-3.
35. Ross JD. Mental health nurse prescribing: the emerging impact. J Psychiatr Ment Health Nurs. 2015;22(7):529-42. doi: 10.1111/jpm.12207.
36. Ross JD, Kettles AM. Mental health nurse independent prescribing: what are nurse prescribers' views of the barriers to implementation? J Psychiatr Ment Health Nurs. 2012;19(10):916-32. doi: 10.1111/j.1365-2850.2011.01872.x.
37. Shannon E, Spence W. The attitudes and views of GPs and physicians to prescribing by heart failure nurse specialists. British Journal of Cardiac Nursing. 2011;6(9):450-5. doi: 10.12968/bjca.2011.6.9.450.
38. Stenner K, Courtenay M. A qualitative study on the impact of legislation on prescribing of controlled drugs by nurses. Nurse Prescribing. 2007;5(6):257-61. doi: 10.12968/npre.2007.5.6.24292.
39. Stenner K, Courtenay M. Benefits of nurse prescribing for patients in pain: nurses' views. J Adv Nurs. 2008;63(1):27-35. doi: 10.1111/j.1365-2648.2008.04644.x.
40. Stenner K, Courtenay M. The role of inter-professional relationships and support for nurse prescribing in acute and chronic pain. J Adv Nurs. 2008;63(3):276-83. doi: 10.1111/j.1365-2648.2008.04707.x.
41. Stenner K, Carey N, Courtenay M. Implementing nurse prescribing: a case study in diabetes. J Adv Nurs. 2010;66(3):522-31. doi: 10.1111/j.1365-2648.2009.05212.x.
42. Stenner KL, Courtenay M, Carey N. Consultations between nurse prescribers and patients with diabetes in primary care: A qualitative study of patient views. Int J Nurs Stud. 2011;48(1):37-46. doi: 10.1016/j.ijnurstu.2010.06.006.
